# Danish study of Non-Invasive testing in Coronary Artery Disease (Dan-NICAD): study protocol for a randomised controlled trial

**DOI:** 10.1186/s13063-016-1388-z

**Published:** 2016-05-26

**Authors:** Louise Nissen, Simon Winther, Christin Isaksen, June Anita Ejlersen, Lau Brix, Grazina Urbonaviciene, Lars Frost, Lene Helleskov Madsen, Lars Lyhne Knudsen, Samuel Emil Schmidt, Niels Ramsing Holm, Michael Maeng, Mette Nyegaard, Hans Erik Bøtker, Morten Bøttcher

**Affiliations:** Department of Internal Medicine, Hospital Unit West, Gl.landevej 61, 7400 Herning, Denmark; Department of Cardiology, Aarhus University Hospital, Aarhus, Denmark; Department of Nuclear Medicine, Hospital Unit West, Herning, Denmark; Department of Radiology, Regional Hospital of Silkeborg, Silkeborg, Denmark; Department of Cardiology, Regional Hospital of Silkeborg, Silkeborg, Denmark; Department of Health Science and Technology, Aalborg University, Aalborg, Denmark; Department of Biomedicine, Aarhus University, Aarhus, Denmark

**Keywords:** Coronary artery disease, Coronary computed tomography angiography, Myocardial perfusion scintigraphy, Cardiac magnetic resonance imaging, Coronary angiography, Fractional flow reserve

## Abstract

**Background:**

Coronary computed tomography angiography (CCTA) is an established method for ruling out coronary artery disease (CAD). Most patients referred for CCTA do not have CAD and only approximately 20–30 % of patients are subsequently referred to further testing by invasive coronary angiography (ICA) or non-invasive perfusion evaluation due to suspected obstructive CAD. In cases with severe calcifications, a discrepancy between CCTA and ICA often occurs, leading to the well-described, low-diagnostic specificity of CCTA. As ICA is cost consuming and involves a risk of complications, an optimized algorithm would be valuable and could decrease the number of ICAs that do not lead to revascularization.

The primary objective of the Dan-NICAD study is to determine the diagnostic accuracy of cardiac magnetic resonance imaging (CMRI) and myocardial perfusion scintigraphy (MPS) as secondary tests after a primary CCTA where CAD could not be ruled out. The secondary objective includes an evaluation of the diagnostic precision of an acoustic technology that analyses the sound of coronary blood flow. It may potentially provide better stratification prior to CCTA than clinical risk stratification scores alone.

**Methods/design:**

Dan-NICAD is a multi-centre, randomised, cross-sectional trial, which will include approximately 2,000 patients without known CAD, who were referred to CCTA due to a history of symptoms suggestive of CAD and a low-risk to intermediate-risk profile, as evaluated by a cardiologist. Patient interview, sound recordings, and blood samples are obtained in connection with the CCTA. All patients with suspected obstructive CAD by CCTA are randomised to either stress CMRI or stress MPS, followed by ICA with fractional flow reserve (FFR) measurements. Obstructive CAD is defined as an FFR below 0.80 or as high-grade stenosis (>90 % diameter stenosis) by visual assessment.

Diagnostic performance is evaluated as sensitivity, specificity, predictive values, likelihood ratios, and C statistics. Enrolment commenced in September 2014 and is expected to be complete in May 2016.

**Discussion:**

Dan-NICAD is designed to assess whether a secondary perfusion examination after CCTA could safely reduce the number of ICAs where revascularization is not required. The results are expected to add knowledge about the optimal algorithm for diagnosing CAD.

**Trial registration:**

Clinicaltrials.gov identifier, NCT02264717. Registered on 26 September 2014.

**Electronic supplementary material:**

The online version of this article (doi:10.1186/s13063-016-1388-z) contains supplementary material, which is available to authorized users.

## Background

An increasing number of patients are referred for evaluation of coronary artery disease (CAD) based on atypical symptoms. No unequivocal diagnostic strategy has been established for diagnosing CAD in patients presenting with symptoms suggestive of stable angina pectoris, and for that reason, clinical practice varies around the world.

The gold standard for detecting hemodynamic obstructive CAD is invasive coronary angiography (ICA) with fractional flow reserve (FFR) measurement [1–3]. However, ICA is costly and involves a small risk of complications and death [4]. Coronary computed tomography angiography (CCTA) has become an established procedure to examine patients with a low to intermediate risk of CAD [5–11]. Due to high negative predictive value, obstructive CAD can be excluded by CCTA in approximately 70–80 % of these patients [11], depending on the risk of the referred population. However, CCTA has consistently proven to have a low positive predictive value, often resulting in an overestimation of the severity of CAD [12], especially in patients with moderate to severe coronary calcification. Consequently, the introduction of CCTA has not led to a substantial decrease in the number of annually performed ICAs or increased the frequency of revascularisation procedures following ICA [13]. The above-mentioned issues raise the question of whether it is possible (1) to make a more precise risk stratification and consequently better selection of patients prior to CCTA and (2) to reduce the number of patients referred for unnecessary ICAs after CCTA.

European guidelines recommend conducting a myocardium perfusion examination after CCTA to reduce the number of ICAs when no revascularization is performed [14], although disagreement still exists regarding which perfusion examination to use.

Cardiac magnetic resonance imaging (CMRI) offers many advantages such as no radiation exposure and high image resolution [15–20]. A lack of scanner capacity and expertise are often mentioned as limitations. Myocardial perfusion scintigraphy (MPS) is an established perfusion test; however, disadvantages such as radiation and low sensitivity are reported as well as a balanced reduction in blood flow to the myocardium in three-vessel disease [21–24]. Many studies assessing the diagnostic accuracy of CMRI and MPS have used ICA diameter stenosis as a reference standard [16, 18[20, 22], which has been shown to be inaccurate in assessing the functional significance of a coronary stenosis compared to ICA-FFR [3].

A meta-analysis on MPS found an average sensitivity of 84 % for women and 89 % for men and a specificity of 78 % for women and 71 % for men using invasive coronary angiography– quantitative coronary angiography (ICA-QCA) as a reference standard [25]. For CMRI, a meta-analysis of studies performed with ICA-FFR as the reference found an average sensitivity of 90 % and a specificity of 87 % [17].

The CADScor System (Acarix A/S, Denmark) is a newly developed technology, which automatically analyses sounds from the heart and extracts multiple acoustic features related to turbulent blood flow, which emerges in a coronary segment with stenosis, and other heart sounds characteristic to CAD [26–29]. Based on these characteristics, the system calculates a score. This score can potentially be used for risk stratification prior to CCTA and in diagnosing CAD [29].

The first objective of the present study is to determine the precision of MPS and CMRI as secondary examinations after CCTA when significant stenosis cannot be excluded.

Our second objective is to examine the diagnostic precision of the CAD score in patients with a low to intermediate risk of CAD and who have been referred to a primary examination by CCTA.

## Methods/design

### Study design

**Fig. 1:**
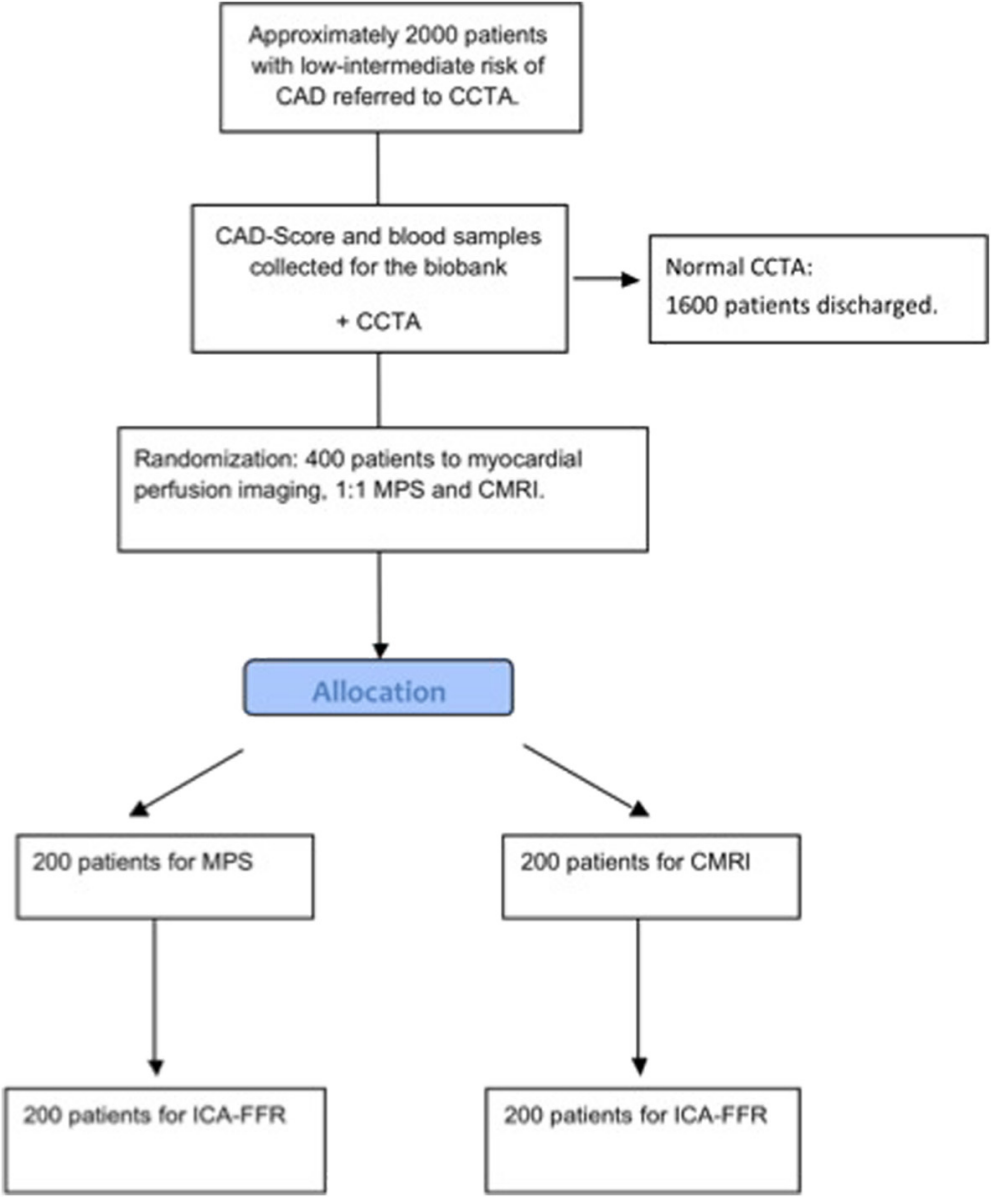
Danish study of Non-Invasive testing in Coronary Artery Disease (Dan-NICAD) patient flowchart. CAD, coronary artery disease; CCTA, coronary computed tomography angiography; CMRI, cardiac magnetic resonance imaging; MPS, myocardial perfusion scintigraphy; ICA-FFR, invasive coronary angiography-fractional flow reserve

Dan-NICAD is an investigator-initiated, multi-centre, randomised, cohort trial examining patients without known CAD, who have been referred to CCTA due to a history of symptoms suggestive of CAD. Study subjects are being recruited at two regional hospitals (Department of Cardiology, Regional Hospitals of Herning and Silkeborg, Denmark). Approximately 2,000 patients with low to intermediate risk of CAD will be included and possibly supplied if needed until a minimum of 300 patients have been randomised and completed perfusion imaging. It is expected that 1,000 patients will be enrolled at each site, as the two centres have similar annual activity in terms of performed CCTAs. Patients with a normal CCTA will not undergo further evaluation, whereas patients with suspected obstructive CAD on CCTA are being randomised. Randomisation is 1:1 for either CMRI or MPS, with subsequent ICA with FFR analysis in both arms. A patient flowchart is included in Fig. 1. All perfusion examinations will be conducted at the two regional hospitals, and the ICA with FFR will be conducted at the Aarhus University Hospital, Aarhus, Denmark. The cohort will be followed for clinical events for 10 years.

### Study population

The cohort consists of patients referred to CCTA with low or intermediate pre-test probability for CAD. The pre-test probability is based on a clinical assessment in an outpatient cardiology setting. Inclusion and exclusion criteria are listed in Table 1. In all enrolled patients, an acoustic examination with the CADScor® system is performed, an interview is conducted, and a blood sample is obtained and stored in a biobank.

**Table 1:** Study enrolment criteria

Criteria for inclusion
- Patients with low intermediate pre-test risk of CAD, with an indication for CCTA
- Qualified patients who have signed a written informed consent form
Criteria for exclusion
- Age below 40
- Pregnant women, including women who are potentially pregnant or lactating
- Contra-indication for adenosine (severe asthma, advanced AV block, or critical aorta stenosis)
- Reduced kidney function, with an estimated glomerular filtration rate (eGFR) < 40 mL/min.
- Contra-indications for MRI
- Allergy to X-ray contrast medium
- Previous PCI, CABG, or POBA

*CAD* coronary artery disease, *CCTA* coronary computed tomography angiography, *MRI*, magnetic resonance imaging, *PCI* percutaneous coronary intervention, *CABG*: coronary artery bypass grafting, *POBA*: plain old balloon angioplasty

### Blood samples

Four blood samples will be collected from all patients before X-ray contrast medium is administered for the CCTA. These samples will be centrifuged, and for each patient, three 1-mL vials of serum, three 1-mL vials of ethylenediaminetetraacetic acid (EDTA) plasma, and three 1 mL vials of heparin plasma are being collected with the purpose of creating a biobank. In addition, the buffy coat layer from both the EDTA-buffered blood and the heparin-buffered blood are being collected and stored for the purpose of DNA and RNA isolation. All samples are being stored in a -80 °C freezer. The purpose of the biobank is to perform genetic analysis related to risk factors and identify new biomarkers associated with the development of arteriosclerosis. The details of these analyses are beyond the scope of this paper.

### CAD score

The acoustic heart score, which is the CAD score, is being measured in connection with the CCTA examination.

Heart sound recordings are obtained with the microphone from the CADScor system (Acarix A/S, Denmark) mounted at the 4th intercostal space just left to sternum using a dedicated patch. The patient has to lie down for 3 min during the recordings and will be asked to hold his/her breath four times for a duration of 7.5 s. As the equipment is sensitive to sounds coming from the outside, tests are conducted in an undisturbed room. Immediately afterward, the recording the equipment will automatically calculate a CAD score. The CAD score is estimated from a fully automated algorithm, which extracts multiple acoustic features from the diastolic heart sounds related to potential turbulence murmurs and other heart sounds characteristics related to CAD [26–29].

The CADScor system analyses the quality of the heart sound; in cases of poor recording quality e.g. a high level of ambient noise, the CADScor device will request a new recording. In these cases, a second attempt is being made to estimate the CAD score.

### Coronary computed tomography angiography (CCTA)

#### Patient preparation

Prior to CCTA, patients are asked to abstain from coffee, tea, and tobacco on the morning of the examination. Ahead of scanning, most patients will be asked to take 50–100 mg of metoprolol or 7.5 mg of ibravadin in order to obtain an optimal heart frequency below 65 beats per minute (BPM). Before imaging, patients with a heart rate higher than 65 BPM will be administered Seloken® (metoprolol tartrate 2.5–20 mg intravenous) if there are no contraindications. Prior to the CCTA, all patients receive 0.8 mg of sublingual nitroglycerin.

#### Imaging protocol

Scans are performed on a 320 multi-slice volume CT scanner (Aquillion One, Toshiba Medical Systems, Japan) using prospective electrocardiogram (ECG) gating.

After anteroposterior and lateral scanograms, a non-enhanced scan will be performed using prospective ECG triggering over a single heartbeat with a gantry rotation and x-ray exposure time of 0.35 s, 0.5-mm slice collimation, tube voltage of 120 kV, and the tube current automatically adjusted with a noise index 80 standard deviation (SD). The start and end positions are determined using the non-enhanced scan. To minimize radiation exposure, the range is reduced as much as possible. In most cases, z-axis coverage is 140 mm but may range from 120 to 160 mm, depending on the length of the heart. The CCTA protocol is performed using a peak tube voltage of 100 kV, except for obese patients where 120 kV is required, a gantry rotation time of 0.35 s, and prospective ECG triggering. 50–80 mL non-ionic contrast agent (Optiray 350 mg/mL, Mallinckrodt, Ireland) is administrated according to patient weight. Real-time bolus tracking is performed in the descending aorta and begins 5 s after the start of the contrast injection. Once a target threshold of 180 Hounsfield units (HU) is reached in the descending aorta, the CCTA will be initiated during the pursuing breath hold.

Data are typically reconstructed at 65–75 % of the RR interval, with supplemental reconstructions when needed. A built-in PhaseExact (Toshiba Medical Systems, Japan) transfers the best phase at slice thickness 0.5 mm to a workstation (Vitrea Advanced Workstation, Vital Images, Minnetonka, MN, USA).

#### Imaging analysis

An experienced cardiologist performs the analyses. An Agatston calcium score is calculated using dedicated software (Vitrea Advanced Workstation, Vital Images, Minnetonka, MN, USA). Luminal diameter stenosis is evaluated in each segment of the coronary tree using the 18-segment model of the Society of Cardiovascular Computed Tomography [9]. Coronary lesions are quantified by visual assessment, and severity is classified as follows: no stenosis—0 % diameter reduction (*≈* 0 % area reduction); mild stenosis—1 to 29 % diameter reduction (*≈* 1 to 50 % area reduction); moderate stenosis—30 to 49 % diameter reduction (*≈* 50 to 69 % area reduction); and severe stenosis—50 to 100 % diameter reduction (*≈* 70 to 100 % area reduction). Severe stenosis and non-evaluable segments with a diameter greater than 2 mm are defined as having obstructive CAD. CCTA is defined as abnormal if obstructive CAD cannot be ruled out in all coronary segments.

### Myocardial perfusion scintigraphy (MPS)

#### Preparation

The patients are advised to abstain from caffeine (coffee, tea, chocolate, and caffeinated medications) 24 h prior to the examination. If possible, patients treated with betablockers are asked to discontinue the treatment 48 h prior to the stress test.

#### MPS protocol

The preferred stress test is a standard symptom-limited exercise test on a bicycle (25 watt/2 min). If the patients are unable to perform an exercise test or if discontinuation of betablocker is contraindicated, a standard adenosine stress test (6-min infusion of 140 μg/kg/min) is performed. If adenosine is contraindicated (allergic asthma or severe COLD defined as a FEV1 < 50 % of expected), patients will be stressed with dobutamine. Tc99m-Sestamibi (10 MBq per kilogram of body weight) is injected at peak exercise or after 3 min of the adenosine infusion, when the target heart rate (= (220 - age) * 0.85) has been reached during the dobutamine infusion. Independent of the stress modality, the heart rate, blood pressure, and ECG are monitored throughout the test.

If the stress images are completely normal, no additional rest study will be performed. If the stress images reveal any suspicion of a perfusion or motion abnormality, a standard rest test (½ h of supine rest followed by injection of 10 MBq Tc99m-Sestamibi per kilogram of body weight) is conducted within 2 weeks.

#### MPS image acquisition

MPS is obtained in the supine position using a dedicated gamma camera (Cardio MD, Phillips Healthcare, Best, Holland) equipped with LEHR collimators and Vantage TM Gadolinium-153 line sources. A step-and-shoot acquisition in 64 projections, 20 s per emission projection, using a 180-degree RAO-LPO orbit and gating in eight frames, is applied. The transmission and emission images are obtained simultaneously. The duration of the transmission scan determines the scan acquisition time based on the Gd-153 source strength; i.e. 20 s per frame for new attenuation correction-sources, increasing to 25 s per frame for older sources (to ensure enough counts in AC image). The energy window is set at 140 keV ± 10 % for the acquisition of the emission images and at 100 keV ± 10 % for the acquisition of the transmission images. An additional 118 keV ± 6 % photopeak window is used to compensate for downscatter of Tc-99 m into the Gd-153 energy window.

After the stress acquisition, a nuclear technician reconstructs the images (see details below), and a nuclear medical physician decides if the patient should undergo a rest test. The raw projection data from the stress and the possible rest acquisitions are stored digitally.

The stress images are obtained within 1 hour after the Tc injection if stress is performed with exercise or adenosine-low exercise and within 3 h after supine adenosine stress or dobutamine stress. The rest images are acquired within 3 h after the injection.

#### Image reconstruction

After obtaining images, the raw image files will be de-identified and processed by an experienced nuclear medical physician using dedicated customized software [30] Cedar Sinai AutoSPECT 3.5 in Phillips Intellispace Portal v6.0.3.12200. An iterative maximum likelihood estimation method algorithm (12 iterations) starting out with a filtered back projection and using a Butterworth analytic filter with a cut off of 0.5 and order of 5.0 will be applied. Motion correction is added if the successive projections move more than 1 pixel in the y axis. Transmission data will be reconstructed similarly except for the use of a cut off of 0.6 and an order of 1.0. After the reconstruction, the left ventricle will be reoriented in short, vertical, and horizontal long axes, and these images will be saved for analysis.

#### Image analysis

**Fig. 2:**
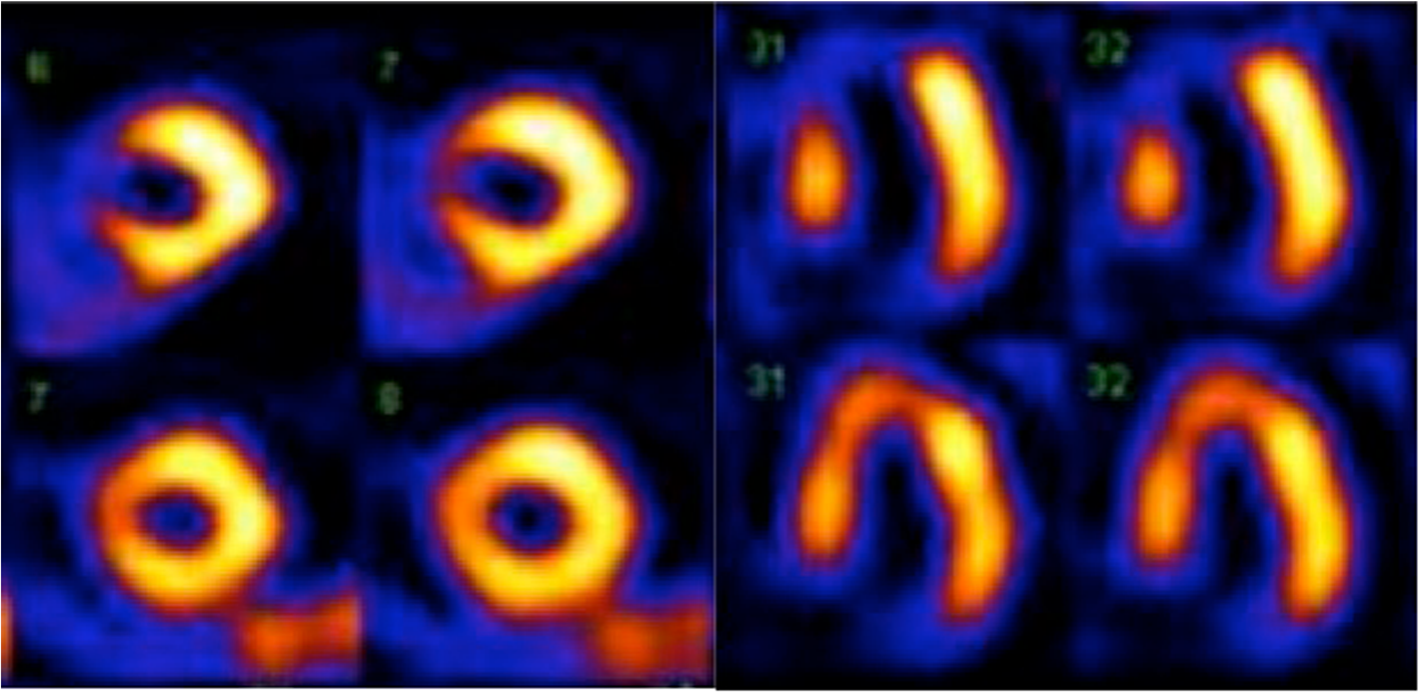
Perfusion defect on myocardial perfusion scintigraphy. Myocardial perfusion scintigraphy with Tc-99 m Sestamibi: short-axis and horizontal long-axis images during stress (upper rows) and rest (lower rows). A severe reversible perfusion defect is seen in the anteroseptal area of the left ventricle

Image analysis will be conducted in Cedar Sinai AutoQuant software in Phillips Intellispace Portal v6.0.3.12200 in an independent core lab by the same nuclear physician, who is blinded to additional patient information and results. The quality of the stress and rest images will be evaluated semi-quantitatively on a visual scale from 1 to 3. Scores defined as 1 represent a good image quality with no artifacts; scores defined as 2 indicate moderate image quality, acceptable for clinical or research diagnosis; and scores defined as 3 are consistent with poor image quality, and diagnosing is impossible due to severe artifacts. The MPS images are assessed using a 17-segment model. For each segment, perfusion defects are scored automatically by the software and adjusted visually on a five-point scoring system (0 is normal, and 4 is the absence of tracer uptake). The scores from the stress and rest images are summed, and a summed stress score (SSS), summed rest score (SRS), and summed difference score (SDS) will be produced. The images will also be evaluated qualitatively with a binary outcome (normal/abnormal). An abnormal MPS scan is defined as (1) an SDS ≥ 43? involving ≥ 2 contiguous segments corresponding to approximately 10 % (reversible ischemia); (2) an SRS ≥ 43? involving ≥ 2 contiguous segments (irreversible ischemia); (3) a combination of reversible and irreversible ischemia involving ≥ 2 contiguous segments (mixed ischemia). Poor image quality (score 3, non-diagnostic) is considered abnormal (Fig. 2).

A hybrid imaging analysis, combined with information from the CCTA and MPS, blinded from the result of the ICA, is performed after the initial analysis.

### Cardiac magnetic resonance imaging (CMRI)

#### Patient preparation and imaging hardware

Patients are advised to abstain from eating at least 2 h before scanning to avoid adverse side effects of the adenosine, which include nausea and vomiting. Patients are also advised to abstain from caffeine 24 h prior to the examination. The CMRI scans will be conducted using two identical 1.5 Tesla systems (Siemens MAGNETOM Avanto, Software release VB17A, Siemens Healthcare GmbH, Germany) using a dedicated 32-channel cardiac-receiver coil. Blood pressure, distal oxygen saturation, and a vector ECG will be monitored continuously during the examinations using a MedRad® Veris® monitoring system (Bayer Healthcare LCC, Berkeley, CA, USA).

All sequences are ECG gated, whereas motion artifacts are minimized by breath-holding and navigator gating. The total scan time is approximately 50 min, which includes patient preparation, scanner set-up, and imaging.

A series of CMRI scans are included to enable the assessment of information on cardiac morphology, function, perfusion, and viability state of the myocardium

#### Imaging protocol

Cardiac morphology is evaluated from axial and sagittal two-dimensional (2D) image series covering the entire heart using a navigator gated single-shot echo-planar fast spin echo sequence (HASTE: Half-Fourier Acquisition Single-shot Turbo spin-Echo) [31] (spatial resolution = 1.9 × 1.3 mm^2^, slice thickness = 8 mm, FOV = 340 mm, TR = 800 ms, TE = 26 ms, Matrix size = 256, flip angle = 160°, Partial Fourier = 5/8, Number of echoes = 123, Time between echoes = 3 ms, and Bandwidth = 698Hz/pixel).

Left ventricular function is evaluated using a time-resolved (cine) 2D balanced Steady State Free Precession (TrueFISP: True Fast Imaging with Steady-state Precession) [32, 33] sequence in which slice planes were acquired yielding four-chamber, three-chamber, and two-chamber views; short-axis views; and an image of the left ventricular outflow tract (LVOT) (spatial resolution = 1.8–2.4 × 1.2–1.6 mm^2^, slice thickness = 8 mm, FOV = 300–400 mm, TR = 201–223 ms, TE = 1.14–1.27 ms, Matrix size = 256, flip angle = 80°, GRAPPA = 3, Bandwidth = 1149 Hz/pixel).

**Fig. 3:**
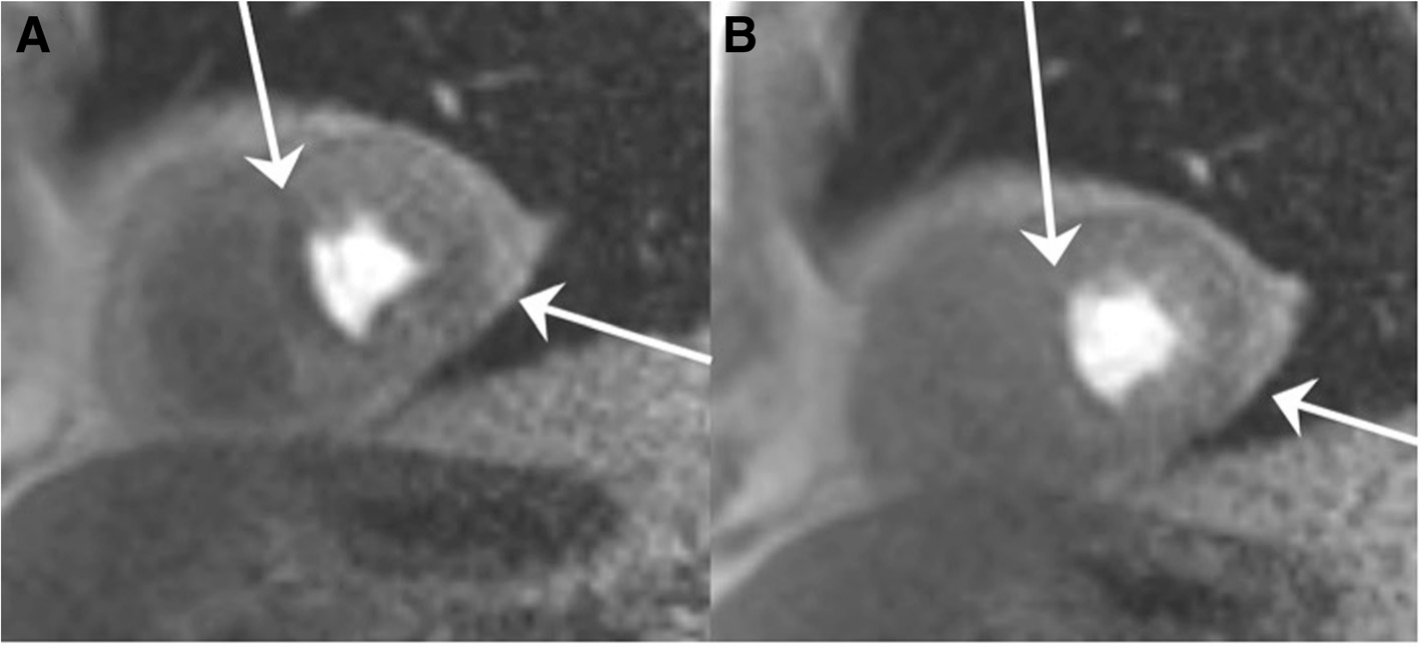
Perfusion defect on coronary magnetic resonance imaging. Coronary magnetic resonance imaging during pharmacological stress induced hyperemia (**a**) and at rest (**b**). The patient has an anteroseptal and lateral reversible perfusion defect in the midcardial segments of the left ventricle

Rest and stress perfusion imaging are performed using an ECG-triggered, contrast-enhanced, dynamic, 2D, gradient, echo, Fast Low Angle Shot (TurboFlash) [34] sequence in three short axis slice planes (basis, mid, and apex) (spatial resolution = 2.4 × 2.1 mm^2^, slice thickness = 10 mm, FOV = 330 mm, TR = 201 ms, TE = 1.05 ms, TI = 130 ms, Matrix size = 160, flip angle = 10°, Partial Fourier = 7/8, GRAPPA = 2, and Bandwidth = 651Hz/pixel). (Fig. 3)

Stress perfusion imaging is carried out after an intravenous bolus injection of either 5 mL of Regadenoson (Lexiscan, Astellas Pharma, USA) or following intravenous adenosine-induced hyperemia. Adenosine is infused for 6 min at 140 μg/kg/min. In case of an inadequate peripheral haemodynamic response to standard dose (an increase in heart rate of more than 10 beats per minute, a systolic blood pressure drop of more than 10 mmHg from baseline or no symptoms), the adenosine infusion is increased to 170 μg/kg/min or even 210 μg/kg/min, depending on the patient response. Independently of the pharmacological stress approach (Regadenoson or adenosine), an intravenous bolus of Gadovist® (Bayer Schering Pharma AG, Germany) or Dotarem® (GD-DOTA, Guerbet LCC, USA) with a concentration of 0.5–0.1 mmol/kg will be administered at a flow rate of 4.0 mL/s through the left cubital vein using a 18 G syringe followed by a 20 mL saline flush (Medrad® Spectris Solaris® power injector, Bayer Healthcare LCC, USA).

Rest perfusion scans are performed after pharmacological stress washout using the same sequence parameters and dose of contrast agent as for the stress perfusion.

Viability evaluation of the myocardium is carried out using a 2D single shot TrueFISP Phase Sensitive Inversion Recovery (PSIR) [35] sequence in the short axis plane of the heart (spatial resolution = 1.8 × 1.8 mm^2^, slice thickness = 8 mm, FOV = 340 mm, TR = 700 ms, TE = 1.09 ms, TI = 300 ms, Matrix size = 192, flip angle = 40°, GRAPPA = 2, Bandwidth = 1447Hz/pixel). The images are acquired approximately 10 min after contrast injection to insure optimal contrast enhancement.

#### CMRI analyses

CMRI analyses are carried out in an independent core lab blinded for additional patient information and results. The image quality of all acquired images is scored qualitatively using a scale from 1 to 3. Scores defined as 1 indicate a good image quality with no artifacts; scores defined as 2 indicate a moderate image quality, which is acceptable for clinical or research diagnosis; and scores defined as 3 indicate poor image quality, and diagnosis is impossible due to severe artifacts. Poor image quality (score 3, non-diagnostic) is considered abnormal.

The images are also evaluated qualitatively with a binary outcome (normal/abnormal) based on the perfusion analyses, presence of wall motion abnormalities, or low ejection fraction. Perfusion analyses at stress and rest are scored as significant, insignificant, or no defect based on the 17-segment model of the American Heart Association. Significant perfusion defects are defined as subendocardial or transmural signal changes in ≥ 2 contiguous segments, corresponding to approximately 6–12 % of the myocardium.

The presence of irreversible defects is considered abnormal if signal changes appear in ≥ 2 contiguous segments on the viability scans. Evidence of regional wall motion abnormalities is scored using a scale from 1 to 4: 1 is normal, 2 is hypokinetic, 3 is akinetic, and 4 is dyskinetic.

A hybrid imaging analysis with combined information from CCTA and CMRI blinded from the result of the ICA are performed after the initial analysis.

### Invasive coronary angiography-fractional flow reserve (ICA-FFR) examination

#### Patient preparation

The ICA examination and the FFR assessments are performed according to present clinical guidelines. The ICA examinations are all conducted at Aarhus University Hospital, Aarhus, Denmark, approximately 4 weeks after referral to CCTA. The ICA is performed though the radial or femoral artery route. Heparin 5000 IE is administered when radial access is used, and 200 μg of nitroglycerine is given intra-coronary after the first angiographic projections.

#### Imaging protocol

All stenoses are visually assessed, and all stenoses above 30 % in vessels with a reference diameter above 2 mm are examined, with at least two projections at least 30 degrees apart revolving around the axis of the vessels. The overlap of vessels is avoided if possible. Angiography is recorded at a time resolution of 15 frames per second (fps).

FFR measurements are performed in all coronary segments with a diameter > 2 mm where ICA shows the presence of stenosis with a diameter ≥ 30 % by visual assessment. The position of the FFR wire is documented angiographically. FFR is measured using a pressure wire (Aeris, St. Jude Medical, Minnetonka, MN, USA) during continuous recording of the pressure curves. Hyperaemia is induced by an intravenous adenosine infusion of a 1 mg/mL concentration of adenosine at 140 μg/L/min. The infusion rate is increased to 200 μg/L/min if a stable FFR value is not achieved. Routine checks are made to ensure that ‘drift’ does not occur after FFR recordings. If the drift value exceeds 1.04 or is below 0.96, the FFR-measurement is repeated. Furthermore, all severe stenoses (> 90 %) in vessels with a reference diameter above 2 mm where there is no immediate indication of treatment, FFR is not possible or not attempted, are noted.

#### Imaging analysis

All evaluations are conducted with assessors blinded to the patient’s CAD score, MPS, or CMRI examinations.

Hemodynamic significant obstructive CAD is defined as FFR ≤ 0.80, or as high-grade stenosis (> 90 % diameter stenosis) by visual assessment in a vessel ≥ 2.0 mm in diameter. If FFR is indicated in a vessel ≥ 2.0 mm but not technically possible, then QCA-based stenosis will be used with a cut-off of ≥ 50 % diameter reduction, which defines an anatomically significant obstructive CAD. The 2D QCA is performed in an independent core lab (ClinFact, Leiden, The Netherlands) using QAngioXA 7.3 QCA software.

### Follow-up

The cohort is followed for 10 years after the CCTA examination. Data are extracted from the Civil Registration System (CRS) and National Patient Registry (NPR) by obtaining diagnosis of CAD including acute coronary syndrome, treatment with PCI/CABG, and causes of death. Cases are verified through electronic medical records that include biochemistry and medication as well as the results of diagnostic examinations.

### Data collection and recordings

All study data are recorded in a secure web-based electronic case record form (eCRF) that enables logging of all data entries (Biostata, Birkerød, Denmark). The CAD score measurement, patient interview, and blood samples are obtained by trained study nurses.

All investigators have access to the eCRF. During the study period, the monitoring of 100 % of the proxy authorizations and informed consent forms and 20 % of the data for biochemistry, Agatston score, comorbidity, and adverse events is conducted by an external contract research organization (Klifo, Glostrup, Denmark). The manuscript has been written following the ‘Standard Protocol Items: Recommendations for Interventional Trials’ and an additional file has been added listing each paragraph and its location in the manuscript (see Additional file 1).

### Randomisation and blinding procedure

Patients are randomised using a randomisation module in the eCRF. An external data manager is responsible for the eCRF, including the randomisation module (Biostata, Birkerød, Denmark). A random allocation sequence is created using a standard computerized random-number generator. The cardiologist analysing the CCTA randomises patients when obstructive CAD is suspected on CCTA. Randomisation is conducted without stratification and in a 1:1 ratio to either MPS or CMRI. The randomisation is open to all investigators and all patients. The physicians performing ICA-FFR are blinded to the results of the MPS or CMRI.

### Data

The following data will be collected during the project and registered in a dedicated eCRF:

- Demography—age, sex, and ethnical origin
- Co-morbidity
- Previous ischemic disease—stable angina pectoris, stroke, and peripheral arterial disease
- Risk factors for ischemic heart disease—genetic disposition, diabetes mellitus, hypertension and hypercholesterolemia, blood pressure, heart rate, weight, height, and smoking status
- Symptoms—typical, atypical, or unspecific chest pain, New York Heart Association (NYHA) functional class, and Canadian Cardiovascular Society (CCS) score system of angina pectoris
- Echo—left ventricle ejection fraction (LVEF) and significant valve disease
- CCTA—scan quality, Agatston calcium score, anatomy, plaque type, and degree of stenosis and radiation exposure
- CAD score—registration of the CAD score, recording time, and positioning of the device
- MPS and CMRI—Scan quality, function, and perfusion defects
- ICA-FFR—data concerning anatomy, localization of stenosis, visual evaluation of stenosis, QCA measurements, FFR measurements, and whether treatment by PCI or CABG is performed
- Follow-up—cardiovascular events as well as possible cardiovascular or other causes of death
- Adverse Events (AE)—all adverse events occurring in the study period are registered

### Endpoints and statistical analysis

Data analysis and reporting will follow the Standards for Reporting of Diagnostic Accuracy (STARD) guidelines and all demographic and baseline characteristics will be presented and analyzed using appropriate statistical methods.

#### First objective

The first objective of the study is to assess the diagnostic accuracy of MPS and CMRI in two comparable samples using hemodynamic significant obstructive CAD at ICA-FFR as the reference standard. The diagnostic performance will be evaluated on a patient level by sensitivity, specificity, predictive values, and likelihood ratios as well as by a binary abnormal/normal test outcome. The sensitivity and specificity of the MPS and CMRI are being compared by Chi^2^ test.

#### Second objective

The secondary objective is to evaluate the diagnostic precision of the CAD score using anatomical significant obstructive CAD at CCTA and ICA-QCA as the reference standard. The diagnostic performance will be evaluated on a patient level as the area under curve receiver operating characteristics (ROC-AUC) with the CAD score as a continuous variable. The CAD score is dichotomized with a positive value above 20, 25, and 30, respectively, and performance is reported with sensitivity, specificity, positive predicative value, negative predicative value, and diagnostic accuracy. In risk stratification the CADScor system operates with three intervals in CAD: < 20, 20–30, and >30 for low, intermediate and high risk, respectively.

Secondarily, the same calculations are made using hemodynamic significant obstructive CAD with ICA-FFR as the reference standard. Comparisons between subgroups are evaluated with ROC-AUC regression analysis. A comparison of the CAD score and MPS or CMRI is evaluated with the McNemars test of sensitivity and specificity at a CAD-score cut-off of 20, 25, and 30.

For all statistical analyses, a two-tailed *p* value < 0.05 was considered statistically significant, and 95 % confidence intervals are being reported when appropriate. Statistical analysis is performed using dedicated statistical software (STATA 13, StataCorp, College Station, TX, USA)

### Sample size

On the basis of previous data, we assumed a sensitivity and specificity of 0.83 for diagnosing obstructive CAD for both MPS and CMRI in patients referred to further examinations after CCTA [15, 17, 22, 36]. Based on these assumptions, a final randomised study cohort of 300 patients is required for a minimum of 10 % absolute precision on both sides (half width of the 95 % confidence interval (CI)) of the expected sensitivity and specificity for both MPS and CMRI. A minimum of 300 patients are needed to achieve sufficient power in the randomised study. To establish this study cohort, we estimate that a primary cohort of approximately 2,000 patients referred to CCTA is needed based on the assumption that 15–20 % of patients need further testing after CCTA.

### Ethical considerations

The study follows the principles outlined in the Declaration of Helsinki. The Danish Data Protection Agency (Case no. 1-16-02-345-14), Danish Health and Medical Authority (Case no. 20140 71252), and the regional committee of Central Denmark engaged in health research ethics (Case no. 1-10-72-190-14) have approved the study protocol. Patients participate in the study only after providing informed written consent both for the randomised part of the study and for their blood samples to be stored in a biobank. The study was registered at ClinicalTrials.gov (Identifier: NCT01344434).

## Discussion

The use of CCTA has proven to be a valid tool for ruling out CAD [6–9] in patients with a low to intermediate pre-test likelihood of CAD. However, the low positive predictive value of CCTA causes ‘unnecessary’ downstream ICA examinations in the sense that no revascularization is required. The need for more specific strategies for non-invasive diagnostic work-up of patients with suspected CAD was demonstrated in a large study comprising 398,978 patients where elective cardiac catheterization showed obstructive CAD in only 38 % of these patients [37]. The Dan-NICAD study aim to test the idea that more patients could potentially avoid unnecessary ICAs by performing a secondary perfusion-imaging test in patients where obstructive CAD could not be excluded with the CCTA. In prior studies looking at low to intermediate pre-test populations, approximately 70 % of the patients did not have obstructive CAD on CCTA [11]. Patients in the Dan-NICAD study in whom obstructive CAD is not excluded by CCTA will be randomised 1:1 to either MPS or CMRI with ICA-FFR as a reference standard for a head-to-head comparison of the diagnostic accuracy of these two modalities.

Several other studies have tested the accuracy of these two techniques. In the CE-MARC study [15], 752 patients with intermediate to high pre-test risks of obstructive CAD underwent both MPS and CMRI with ICA-QCA as a reference standard. The study showed both higher sensitivity and specificity of CMRI compared to MPS. The MR-IMPACT study [38] also made a head-to-head comparison of the two techniques and showed superiority of CMRI. Here, the patients were highly selected. Patients had either undergone an ICA (positive or negative) or had a positive MPS and were scheduled for ICA. Both studies used ICA-QCA, a reference standard proven not to be ideal [3], as it compares functional tests with an anatomical test. ICA-QCA is shown to overestimate the severity of CAD compared to FFR [3]. The MARCC study [11], with a population of low-to intermediate pre-test probability, tested the combined use of CCTA and CMRI for the diagnostic evaluation of patients with suspected CAD using ICA-FFR as a reference standard when disease was suspected on either CCTA or CMRI. The study showed improved specificity and diagnostic accuracy using a combined set-up.

As the availability of CMRI is limited in most clinical settings, we chose a stepwise approach with CCTA as the initial examination based on the high negative predictive value of this approach. [11]. The CAD score combined with the Diamond-Forrester score compared to Diamond-Forrester score alone has previously been shown to improve the risk assessment in patients suspected of stable CAD [29]. Optimizing the risk assessment might reduce the use of more advanced diagnostic testing and can potentially serve as an easy, safe, and low-cost supplement in diagnosing CAD.

### Perspective

Despite the advances in knowledge and research within the field of CAD large variations still exist in the diagnostic approach for this large group of patients. The effect of a secondary perfusion imaging examination after CCTA in a group of patients with low to intermediate pre-test probability of CAD could potentially reduce the number of unnecessary ICAs where no revascularisation is required. The results of this study are expected to make an important contribution to the improvement of diagnostic strategies for patients suspected of CAD.

## Trial status

The trial is ongoing. The first patient was enrolled on 11 September 2014. As of September 2015, 1,100 subjects have been enrolled, and 226 subjects (23.5 %) have been randomised. Enrolment completion is expected in May 2016.

## Abbreviations

BPM, beats per minute; CABG, coronary artery bypass graft surgery; CACS, coronary artery calcium score; CAD, coronary artery disease; CCS, Canadian Cardiovascular Society; CCTA, coronary computed tomographic angiography; CI, confidence interval; CMRI, cardiac magnetic resonance imaging; CRO, contract research organization; CRS, Civil Registration System; ECG, electrocardiogram; eCRF, web-based case record form; EDTA, ethylene diaminetetraacetic acid; fps, frames per second; ICA, invasive coronary angiography; ICA-FFR, invasive coronary angiography-fractional flow reserve; LVEF, left ventricle ejection fraction; MPS, myocardial perfusion scintigraphy; NPR, National Patient Registry; NYHA, New York Heart Association; PCI, percutaneous coronary intervention; QCA, quantitative coronary angiography; ROC, receive operator characteristics; SD, standard deviation; SDS, summed difference score; SSS, summed stress score; STARD, Standards for Reporting of Diagnostic Accuracy; 2D, two-dimensional; 3D, three-dimensional

## Additional file

Additional file 1: SPIRIT checklist. Each SPIRIT checklist paragraph has been addressed regarding location in the manuscript. In cases where the paragraph is not relevant to this manuscript it has been addressed with not applicable. (PDF 117 kb)
